# A Prospective Cohort Evaluation of a Robotic, Auto-Navigating Operating Microscope

**DOI:** 10.7759/cureus.662

**Published:** 2016-06-30

**Authors:** Michael A Bohl, Mark E Oppenlander, Robert Spetzler

**Affiliations:** 1 Department of Neurosurgery, Barrow Neurological Institute

**Keywords:** microneurosurgery, operating microscope, auto-positioning, robotics

## Abstract

The unique challenges inherent to microneurosurgery demand that we stay on the forefront of new surgical technologies. Many believe the next major technological advance in neurosurgery will be the widespread application of image-guided robotics in the operating room. We evaluated a novel technology for image-guided robotic auto-navigation of the operating microscope in a prospectively enrolled cohort of patients.

Twenty patients were prospectively enrolled for analysis. Data were collected on the extent of resection, operative time, estimated blood loss, time taken to set up the new software, and complications encountered. Software accuracy, reliability, and usefulness in the case were subjectively evaluated.

The most commonly  treated pathologies were cavernous malformation (n = 5), arteriovenous malformation (n = 4), and meningioma (n = 4). The time to set up the new software interface before the start of the operation was <60 seconds in all cases. Subjective evaluation in each case revealed the robotic interface to be accurate, reliable, and useful. The new technology was significantly more useful in deeper lesions.

The addition of image-guided robotic auto-positioning features to the operating microscope has a great potential to advance the field of neurosurgery. This study is the first prospective evaluation of such a technology in a patient cohort. The results suggest that the newest robotic auto-positioning technology has the potential to improve the neurosurgeon's efficiency and efficacy, thereby positively impacting patient safety and surgical outcomes, especially in cases involving deep-seated lesions.

## Introduction

From the development of surgical electrocautery to the introduction of the operating microscope, the field of neurosurgery has historically been a catalyst for collaborative advances in surgical and engineering technology [1-3]. Such collaboration has, in turn, advanced the neurosurgeon’s operative ability. The wedding of neuronavigation technology to the operating microscope, for example, has advanced the field of microneurosurgery to such an extent that deep-seated lesions previously considered untreatable are now commonly visualized and resected [4-6]. The unique technical challenges inherent to microneurosurgery demand that neurosurgeons stay on the forefront of new surgical technologies. Many believe that the next major technological advance will be the introduction of robotics to the neurosurgical operating room [7, 8].

Recently, several groups have tested different robotics systems for use in both cranial and spinal neurosurgical procedures [9-16]. One particularly interesting development is the introduction of robotics to the operating microscope to create a self-navigating microscope capable of automated positioning and surgical approach finding [13]. This technology has several potential advantages, including smoother and more accurate positioning of the microscope, less disruption to the operative workflow for microscope positioning, and the ability to automatically acquire intracranial targets and surgical approaches at any time in the course of a procedure, even prior to target visualization. All these advantages have the potential to make surgery on deep intracranial targets more efficient, more controlled, and safer for the patient. However, a state-of-the-art robotic auto-positioning microscope has yet to be evaluated intraoperatively to compare the utility of this technology to a standard, manually positioned microscope. The purpose of this study was to provide the first clinical evaluation of modern robotic auto-navigation technology in a self-controlled, prospectively enrolled cohort of patients.

## Technical report

### Methods

Description of the Evaluated Technology

The robotic auto-navigation system evaluated in this study was produced via a collaborative effort between a neuronavigation company (Medtronic Navigation, Louisville, Colorado) and a surgical microscope company (Carl Zeiss Meditec AG, Oberkochen, Germany). This collaborative effort produced a user interface with three different options for automatic positioning: AutoLock Current Point, Align Parallel to Plan, and Point to Plan Target. AutoLock Current Point allows the surgeon to lock onto a target defined by the microscope’s position and focal length during surgery. The Align Parallel to Plan feature aligns the microscope’s trajectory parallel to a predefined surgical plan (as defined by pre-selected entry and target points using the neuronavigation software). The Point to Plan Target option focuses the microscope on a predefined target. Oppenlander et al. described these features in a descriptive article of an earlier version of this technology [17]. The setup of the auto-navigation system requires very few additional steps to the setup of a conventional manual navigation system. After the patient’s head is registered to the intraoperative imaging guidance system in the usual fashion, an operating microscope equipped for intraoperative navigation is brought into the surgical field. Surgical targets can be selected either preoperatively or intraoperatively using the robotics software package. The only setup that is required in addition to a conventional navigation system is the selection of surgical targets using either the StealthStation (Medtronic, plc, Dublin, Ireland) imaging (typically done preoperatively) or the focal point of the operating microscope (done intraoperatively when visualizing the target).

The most recent version of the auto-navigation software (Cranial software v.2.2.7) includes a function for toggling the AutoLock feature on and off simply by pressing the StealthStation foot switch, giving the surgeon better freedom in switching back and forth between manual and auto-navigation modes. The newest software also enables the surgeon to AutoLock to the target point of a predefined plan. Previously, the user could AutoLock only to a point that was defined at that time by the microscope’s focal length, unrelated to a pre-defined target or trajectory.

Evaluation of the Technology

Twenty consecutive patients scheduled to undergo resection of an intracranial vascular or neoplastic lesion were enrolled for participation in this study. The robotic auto-navigation system was evaluated intraoperatively by the senior author, who operated using both manual navigation and auto-navigation modes relatively equally throughout each case so that fair comparisons could be made between these two methods of microscope navigation. The performance of the robotic auto-navigation technology was compared in each case to that of manual navigation mode. Children (age <18 years), pregnant women, and prisoners were excluded from the study. All patients were treated at Barrow Neurological Institute. The Institutional Review Board of St. Joseph’s Hospital and Medical Center in Phoenix, Arizona, approved this study.

Evaluation consisted of several prospectively collected variables, including the extent of resection (EOR), operative time, estimated blood loss (EBL), time taken to set up the new software (entailing the neurosurgeon selecting the predicted entry and target points on the StealthStation prior to preparing and draping the patient), complications encountered, lesion depth, and a prospectively collected subjective evaluation of the software’s accuracy, reliability, and usefulness as compared to manual navigation for each case. Lesion depth was scored as convexity access (CA), deep surface access (DSA), or deep intraparenchymal access (DIP). The senior author alone was responsible for scoring the software’s accuracy, reliability, and usefulness for each case. Scoring was done on a scale of one to five, with a score of one indicating manual navigation is superior to robotic auto-navigation, a score of three indicating manual and auto-navigation modes are equally useful, and a score of five indicating auto-navigation is superior to manual navigation.

Basic statistical analyses for the entire cohort as well as comparisons of lesion depth subgroups were performed using the independent-samples t-test. A P value < 0.05 was considered significant.

### Results

Twenty patients were enrolled in the study and underwent surgery over a 6-week period. The cohort included seven men and 13 women. Patients’ mean age was 49.7 years (range, 25–69 years). Lesions treated were cavernous malformation (n = 5), arteriovenous malformation (n = 4), meningioma (n = 4), vestibular schwannoma (n = 2), craniopharyngioma (n = 1), epidermoid (n = 1), glioblastoma (n = 1), hemangioblastoma (n = 1), and juvenile pilocytic astrocytoma (n = 1). The surgical approaches were modified orbitozygomatic (n = 4), pterional (n = 4), retrosigmoid (n = 3), suboccipital (n = 3), occipital (n = 2), parieto-occipital (n = 1), frontotemporal (n = 1), interhemispheric (n = 1), and parieto-occipital transventricular (n = 1). Gross total resection was achieved in 18 patients, and two patients underwent planned subtotal resections. The mean intraoperative blood loss was 292.5 mL (range, 50–2000 mL). The time required to set up the StealthRobotics interface before the start of the operation was <60 seconds in all cases. No operative complications were noted. Ten lesions were designated as DIP, and five each were classified as DSA and CA. Table 1 summarizes the patient demographics and the recorded objective variables.

**Table 1:** Patient demographics and objective variables AVM, arteriovenous malformation; CA, convexity access; DIP, deep intraparenchymal access; DSA, deep surface access; EBL, estimated blood loss; EOR, extent of resection; GTR; gross total resection; JPA, juvenile pilocytic astrocytoma; mOZ, modified orbitozygomatic; OR, operating room; STR, subtotal resection; STR*, planned subtotal resection. *Items were scored on a scale of 1 to 5. Mean values were 4.3 (accuracy), 4.7 (reliability), and 3.7 (usefulness).

Case #	Age (Y)	Sex	Pathology	Approach	Setup Time (s)	Intraoperative Complications	Lesion Access	Subjective Score*		
								Accuracy	Reliability	Usefulness
1	55	M	Hemangioblastoma	Suboccipital	<60	None	DIP	5	5	3
2	69	F	Meningioma, incisural and falcine	Parieto-occipital, trans-ventricular	<60	None	DIP	5	5	5
3	59	F	Epidermoid, suprachiasmatic	mOZ	<60	None	DSA	5	5	2
4	52	F	Craniopharyngioma, recurrent	mOZ	<60	None	DIP	5	5	4
5	68	F	Temporal cavernous malformation	Pterional	<60	None	DIP	4	5	4
6	45	M	Parieto-occipital AVM	Parieto-occipital	<60	None	DIP	5	5	5
7	45	F	Cerebellar AVM	Suboccipital	<60	None	CA	4	4	3
8	49	M	Inferior frontal cavernous malformation	Pterional	<60	None	DIP	4	4	4
9	60	F	3rd ventricular cavernous malformation	Inter-hemispheric	<60	None	DIP	5	5	5
10	65	F	Glioblastoma, recurrent	Frontotemporal	<60	None	CA	3	3	3
11	48	M	Frontal cavernous malformation	mOZ	<60	None	DIP	4	5	5
12	59	F	Occipital AVM	Occipital	<60	None	DIP	4	5	4
13	30	M	Vestibular schwannoma	Retrosigmoid	<60	None	DSA	4	5	2
14	55	F	AVM, recurrent	Pterional	<60	None	CA	4	5	3
15	25	F	Cerebellar JPA	Suboccipital	<60	None	DIP	4	5	4
16	38	F	Occipital AVM	Occipital	<60	None	CA	4	4	3
17	54	F	Vestibular schwannoma	Retrosigmoid	<60	None	DSA	5	5	3
18	26	F	Petrotentorial meningioma	Retrosigmoid	<60	None	DSA	4	5	4
19	44	M	Temporal convexity meningioma	Pterional	<60	None	CA	4	5	3
20	48	M	Sphenoid wing meningioma	mOZ	<60	None	DSA	4	4	4

The senior author’s subjective feedback on the system’s accuracy, reliability, and usefulness is summarized in Table 1. This assessment revealed a mean accuracy score of 4.3/5, a reliability score of 4.7/5, and a usefulness score of 3.7/5. Lesion depth subgroup analysis revealed that in the DIP subgroup, the technology had an accuracy score of 4.5, a reliability score of 4.9, and a usefulness score of 4.3 (Table 2). In the DSA subgroup, the accuracy score was 4.4, the reliability score was 4.8, and the usefulness score was 3.0. The CA subgroup had an accuracy score of 3.8, a reliability score of 4.2, and a usefulness score of 3.0. Comparison of these subgroups revealed statistically significant differences between the scored accuracy in DIP vs CA lesions (P = 0.02), as well as the usefulness in DIP vs DSA (P = 0.01) and DIP vs CA (P < 0.001) lesions.

**Table 2:** Comparison of lesion depth subgroup scores* CA, convexity access; DIP, deep intraparenchymal access; DSA, deep surface access. ^*^Items were scored on a scale of 1 to 5. ^†^Bold values indicate statistically significant differences.

	DIP (Mean Score)	DSA (Mean Score)	CA (Mean Score)	P value^†^
				DIP vs DSA
				DIP vs CA
				DSA vs CA
Accuracy	4.5	4.4	3.8	P = 0.74
				P = 0.02
				P = 0.09
Reliability	4.9	4.8	4.2	P = 0.63
				P = 0.13
				P = 0.20
Usefulness	4.3	3.0	3.0	P = 0.01
				P < 0.001
				P > 0.99

## Discussion

The 20 patients in whom intraoperative testing of the new software was conducted, harbored a wide spectrum of pathologies ranging from benign to malignant, and superficial to deep. They were treated via a variety of surgical approaches. The EOR, EBL, and type and number of complications were all consistent with those typically achieved by the senior author for similar cases and treated using manual navigation only [18-20]. The operative time was also consistent with typical operative times for cases of correlating complexity, although the interpretation of these numbers is complicated by the fact that in each case substantial contribution was made at opening and closing by different residents or cerebrovascular fellows of presumably different skill levels. Of note, the additional time taken to set up the software was less than one minute in all cases, and simply entails the neurosurgeon selecting predicted entry and target points on the StealthStation prior to preparing and draping the patient. We believe the minimal setup time for the new technology to be of great benefit by allowing automated maintenance of microscope trajectory and focus on the intended target.

Notably, no intraoperative complications occurred while incorporating the new technology into the operative work flow. Complications that might be encountered as a direct result of this technology include the possibility of the microscope coming into contact with the patient during auto-positioning. However, since the greatest risk would be associated with the microscope descending in the vertical axis, this is not a realistic risk because changing the vertical axis is only performed by adjusting the focal length, not the physical position of the microscope. The automatic movements made by the microscope during target acquisition are only angular and are typically very small, making the possibility of patient contact during auto positioning extremely unlikely. Inadvertent or unrecognized loss of navigation accuracy is also a potential complication of this software. Although this problem was not encountered in the present cohort, it is possible that surgeon contact with the microscope during automatic repositioning, movement of the cranial frame in relation to the patient’s head, or loss of visualization of the cranial or microscope frames by the neuronavigation receiver could cause the introduction of error in navigation accuracy. Complications resulting from lost navigation accuracy can be avoided by ensuring that these missteps do not occur, and also by maintaining anatomical orientation during the case such that one is able to continually perform mental checks of the navigational accuracy. Overall, this study demonstrates a favorable safety profile when this technology is incorporated into the operating room.

The subjective evaluation of this technology further indicates that robotic auto-positioning may be found in certain cases to be superior to manual navigation. When the above cohort is considered as a whole, subjective scoring suggests that this new technology is moderately more useful than manual navigation. The lesion depth subgroup analysis further reveals that as lesions trend from superficial (CA subgroup) to deep (DIP subgroup), the accuracy, reliability, and usefulness of this technology over manual navigation increases. This conclusion is further supported by the significant difference found in subjective scoring of the software’s usefulness in DIP compared to either DSA or CA lesions. The increased accuracy of the software for surgery on deeper lesions is likely because less brain shift occurs in deeper locations, which is also likely to have affected the user’s subjective rating of usefulness. Overall, these results suggest that this technology is best suited for deep-seated lesions in eloquent areas, and that the integration of this new technology into the operating room has the potential to increase operative efficacy and safety.

### Limitations

This study is limited by its small size, a single-surgeon subjective scoring system used for clinical evaluation, and the lack of a control group for comparison. These limitations were considered acceptable, however, given that this was intended to be a pilot study into the potential utility of the described software. Further investigations into the utility of this technology and its effect on surgical outcomes would ideally include a larger population of patients with more homogeneous lesion characteristics.

## Conclusions

Technological advances in both the operating microscope and neuronavigation have revolutionized the field of neurosurgery. Finding new, inclusive methods for improving these technologies is likely to further advance our field. This study is the first prospective evaluation of the clinical utility and safety of a robotic auto-positioning technology in a patient cohort. The results suggest that this technology has the potential to improve the neurosurgeon's efficiency and efficacy, thereby positively impacting patient safety and surgical outcomes, especially in cases involving deep-seated cranial lesions.
